# What constitutes victims of toxicity - identifying drivers of toxic victimhood in multiplayer online battle arena games

**DOI:** 10.3389/fpsyg.2023.1193172

**Published:** 2023-06-16

**Authors:** Bastian Kordyaka, Samuli Laato, Sebastian Weber, Bjoern Niehaves

**Affiliations:** ^1^Working Group Digital Public, Faculty 3 - Mathematics and Computer Science, University of Bremen, Bremen, Germany; ^2^Gamification Group, Tampere University, Tampere, Finland

**Keywords:** toxic behavior, toxicity, multiplayer online battle arena games, victims of toxicity, online disinhibition effect, social cognitive theory, theory of planned behavior, League of Legends

## Abstract

**Introduction:**

Toxic behavior (i.e., toxicity) is a pervasive problem in online gaming communities such as League of Legends. This issue arises from factors such as frustrating and stressful in-game experiences and online disinhibition. Prior research on addressing toxicity has focused primarily on the perpetrators and how to mitigate their negative behavior and the consequences. The aim of this study was to approach toxicity from the perspective of the victims instead, and consequently, to investigate the factors that contribute to the experience of victimhood in multiplayer online battle arena games.

**Methods:**

A global sample of League of Legends and Defense of the Ancients 2 players (*n*=313) was collected to test hypotheses based on three theoretical approaches drawn from previous work, namely, the online disinhibition effect, social cognitive theory and theory of planned behavior. Participants were asked to complete a survey that included variables related to the three theoretical approaches.

**Results:**

The results of the study indicated that self-efficacy, and benign and toxic disinhibition, were the most relevant antecedents for the experience of being a victim of toxicity. Accordingly, the findings thus suggest that players with low self-efficacy and high online disinhibition may be more likely to experience victimhood in multiplayer online battle arena games. In general, insights based on our study demonstrate that individual characteristics partially explain why some players are more susceptible to toxic behavior than others.

**Discussion:**

The study’s results have practical implications for game developers and policymakers, particularly in the areas of community management and player education. For example, game developers may consider incorporating self-efficacy training and disinhibition reduction programs into their games. Overall, this study contributes to the growing body of literature on toxicity in online gaming communities and invites further research into toxicity from the perspective of the victims.

## 1. Introduction

One of the big technological disruptions of the 21st century has been the rise of multiplayer online gaming, and one of the popular and most rapidly growing genres of these games are multiplayer online battle arena (MOBA) games (Argenio, 2018; Mora-Cantallops and Sicilia, 2018). Manifestations related to this socio-technological disruption are the occurrences of (mostly multiplayer) MOBAs such as League of Legends (LoL), Defense of the Ancients 2 (DOTA 2), or Heroes of the Storm and their cultural relevance and economic success. As an example, current estimations suggest that League of Legends (e.g., one of the most relevant MOBA game titles at the moment) had up to 180 million monthly players in June 2022 and generated $1.63 billion in 2021 (LeagueFeed, 2022), which are numbers that are steadily growing. Furthermore, League of Legends already has its own Netflix series called Arcane that is broadcasted on Netflix and Twitch (Polhamus, 2021) and universities across the globe already offer scholarships related to the game (Sabtan et al., 2022). Summarizing, MOBAs can be considered one of the most relevant building blocks of digital cultures and entertainment.

Looking at the unique features of successful MOBA titles at the moment, two characteristics are standing out (a) real time interaction and (b) (multiplayer) competition that enable new forms of player experiences (Hamari and Sjöblom, 2017; Adinolf and Turkay, 2018). However, consequences of this technological innovation can be classified into two broad categories. First, positive outcomes such as increased player motivation and enjoyment represent the bright side of this playful disruption (Kim and Shute, 2015). Second, and opposed to this, new forms of negative phenomena became apparent presenting the dark side of the dissemination of MOBAs (Blackburn and Kwak, 2014; de Mesquita Neto and Becker, 2018). One such instance related to the dark side, is toxic behavior describing various negative actions during gameplay including criticizing, harassment, flaming, trolling, and cheating others (Adinolf and Turkay, 2018). Interestingly, the majority of toxic behavior is targeted toward members of the own team. Despite several attempts of the industry to reduce the probability of the occurrence of toxic behavior (TB) offering several reporting features, it is still a serious problem and considered to be one of the main drivers of the exodus of players in a variety of MOBAs (Kordyaka and Kruse, 2021).

Previous research already addressed several aspects related to toxic behavior such as (a) deriving a validated measurement instrument (Kordyaka et al., 2019), (b) proposing a unified theory of the occurrence of toxicity perpetration (Kordyaka et al., 2020), (c) showing relationships to related constructs such as loneliness or well-being (Mandryk et al., 2020), (d) illustrating the normalization as part of the game culture (Beres et al., 2021), and several more granular insights regarding differences of game and player characteristics. However, consulting the theoretical origins of toxic behavior in cyberbullying research, one aspect neglected up to now is the differentiation between the roles of perpetrators (i.e., actively exerting toxicity toward others) and victims of toxic behavior (i.e., becoming the target of the toxicity of others) (Vandebosch and Van Cleemput, 2009; Bastiaensens et al., 2014). Most previous research took on the stand of perpetrators of toxic behavior neglecting the complementary perspective of victims. Consequently, it is still unclear what empirical patterns the experience of being a victim of toxicity follows and how this interacts with perpetrators.

Answering this, the present paper aims to close this gap by better understanding victimhood of toxicity. For this, we build on previous work showing that online disinhibition effect (ODE), social cognitive theory (SCT), and theory of planned behavior (TPB) are suitable theoretical approaches to explain toxic behavior perpetration (Kordyaka et al., 2020). As contexts for our study, we refer to two of the most successful MOBAs at the moment League of Legends and Defense of the Ancients 2 to have the chance to comprehend indicators of the external validity, of our findings while controlling for differences between games. Methodologically, we apply covariance-based statistics (e.g., regression analyses and structural equation modeling) and a digital survey approach to collect data using Amazon Mechanical Turk (MTurk). Accordingly, our paper is guided by the subsequent research question (RQ):

*RQ*: What variables informed by online disinhibition effect, social cognitive theory, and theory of planned behavior predict the experience of becoming a victim of toxicity in multiplayer online battle arena games?

To answer our RQ, we seek to expand existing research to the topic of the experience of being a victim of toxicity. For this purpose, the procedure of our study is guided as follows. First, we introduce the related work necessary to understand the theoretical background and specify hypotheses. Following this, we present the methodology and derive the results to test our hypotheses. Afterward, we discuss the implications of our findings and close with a short conclusion. With our study, we want to make several contributions. Firstly, implications will allow academia to better understand an additional aspect related to the occurrence of toxicity (namely the experience of being a victim of toxicity), which will provide a variety of resulting research opportunities. Secondly, this study provides practical implications with the opportunity for the industry to better understand and curb toxicity, and avoid player turnover while improving the overall game play experience for players.

## 2. Related work

### 2.1. Multiplayer online battle arena games

During the last decades enabled by the technological advancements, new forms and genres of video games have emerged. One particularly salient and relevant manifestation of this MOBAs representing a fusion of existing and older game genres such as action, role-playing, and strategy video games (Ferrari, 2013; Johnson et al., 2015). The market of MOBAs include globally successful and well-known game titles such as League of Legends or Defense of the Ancients (Yang et al., 2014; Mora-Cantallops and Sicilia, 2018). Due to their economic success and worldwide dissemination, MOBAs are one of the spearheads of the digital culture of the younger generations (Bányai et al., 2019). On a level of manifestations of the relevance of MOBAs, League of Legends as an example, already disposes an action-adventure streaming series called Arcane enjoying great demand on Netflix (Liu et al., 2022).

MOBAs possess several unique characteristics related to their gameplay that increase their disposition for toxic behaviors. Thus, they are highly dynamic, competitive, and frustrating, while cultivating less autonomy compared to older multiplayer online games (Johnson et al., 2015). As defaults, every MOBA player controls a single champion in one of two teams consisting of five players each with different abilities. The goal of the game is to destroy the others team Nexus. For this, players can earn experience points to level up their champions and gold to buy items increasing the abilities of their champions. Opposed to older games, all players involved start with the exact same amount of experience and gold, and there is no possibility to have any advantage investing money. Depending on the outcome of the most frequently played game mode ranked, each player wins or loses points representing their overall skill level. During games collaborating and communicating with others is key to victory. For this, players predominantly use text chat and ping commands (describing player-relayed alerts that provide gameplay information to the entire team) as communicative sources. Taken together, based on their characteristics, MOBAs are a particularly suitable stage for the occurrence of toxicity.

### 2.2. Experience of becoming a victim of toxicity

To theoretically capture the experience of being a victim of toxicity, we refer to previous work regarding toxic behavior perpetration. Originated in work related to cyberbullying research (Extremera et al., 2018; McLoughlin et al., 2020; Zhao and Yu, 2021), toxic behavior possesses several unique characteristics such as a much more temporary duration happening in real-time, not necessarily intentional and rather a spontaneous attempt to cope with negative in-game scenarios (Chen et al., 2017; Kordyaka et al., 2020, 2023). Furthermore, the majority of corresponding behaviors are directed toward teammates (Adinolf and Turkay, 2018). Following this, a definition widely used for toxic behavior perpetration originates from Neto et al. (2017) who understand toxicity as an umbrella term to capture different negative behaviors (such as harassment, flaming, trolling, and criticizing others) that occur when a player comes across a negative event during a game corroding team effort, harming the game ambiance, generating anger and frustration, leading to contaminated, and disseminated toxic type of communication while playing. Regularly, toxic behavior is directed toward members of the own team and can be understood as an attempt to externally attribute negative incidents during a game (Kordyaka and Kruse, 2021). The most common forms of toxicity are flaming (e.g., insulting others often including profanity or other offensive language in the chat) and trolling (e.g., causing discord in other players), which occurs in almost every (ranked) game, and therefore, substantially narrows the gameplay experience (Adinolf and Turkay, 2018).

Previous research already identified relevant antecedent variables of toxic perpetration, such as toxic disinhibition, attitude, and behavioral control and consequences, such as deteriorated team performance and cohesion (Kordyaka et al., 2020; Kowert, 2020; Monge and O’Brien, 2022). However, to the best of our knowledge, no study up to now has explored the experience of being a victim of toxicity in the context of MOBAs, which is surprising because the occurrence of toxicity is always an interaction of at least two different player roles. For the purposes of our study, we understand victimhood of toxicity as a negative situation during a game in which a player becomes the victim of toxic behaviors of others such as criticism, harassment, responsibility diffusion, flaming, trolling, or cheating. Consulting previous research in the context of MOBAs, only one study derived quantitative indicators of the relationship between victimhood of toxicity and toxic behavior perpetration indicating a (fully mediated) positive relationship between both variables (Kordyaka et al., 2020) suggesting a substantial overlap in roles of perpetrators and victims of toxicity.

### 2.3. Understanding the experience of being a victim of toxicity

To better understand the experience of being a victim of toxicity, we subsequently introduce theoretical approaches that already showed its potential to explain toxic behavior perpetration.

#### 2.3.1. Online disinhibition effect

The online disinhibition effect describes the lack of restraint an individual feels when communicating online in comparison to communicating in-person (Lapidot-Lefler and Barak, 2012; Cheung et al., 2016; Lowry et al., 2016). Furthermore, the online disinhibition effect postulates two dimensions of disinhibition (a) benign disinhibition (describing behaviors helping someone and showing kindness) and (b) toxic disinhibition (describing behaviors such as rude and violent language). Previous studies already showed that individuals involved in negative digital behavior exhibited higher levels of disinhibition (Udris, 2014) and that the perceived level of anonymity facilitates such behaviors (Lowry et al., 2016, 2017). Additionally, toxic disinhibition and benign disinhibition both showed a distinct positive relationship (Udris, 2014). For the purposes of our study, we argue that the online disinhibition effect is a well-suited approach to capture the technological environment of MOBAs, due to the high levels of anonymity present.

Existing research within the context of MOBAs only partially addressed the concept of disinhibition and only in relation to the role of toxic behavior perpetration. Nonetheless, one study already showed that toxic disinhibition was the most relevant antecedent variable of toxic behavior perpetration (Kordyaka et al., 2020), whereby benign disinhibition did not reach any statistical significance. We build on this, aiming to extend previous research related to the online disinhibition effect to the MOBA context. Based on the arguments knowing that (a) both forms of disinhibition are positively correlated and (b) toxic disinhibition is a positive predictor of toxic behavior perpetration, we argue that this should be similarly the case for victimhood of toxicity as well. Based on this, we specify our first two hypotheses:

*Hypothesis ODE.1*: Benign disinhibition has a positive influence on toxicity victimhood.

*Hypothesis ODE.2*: Toxic disinhibition has a positive influence on toxicity victimhood.

#### 2.3.2. Social cognitive theory

The social cognitive theory is a learning theory positing that learning occurs in a social context with a dynamic and reciprocal interaction of the individual, the environment, and resulting behaviors (Bandura, 1986, 2002). The unique feature of the social cognitive theory is the emphasis on social influence and external and internal reinforcement postulating that individual learn either through direct experience or through observation (Luszczynska and Schwarzer, 2015). For the purpose of our paper, we follow a conceptualization of the social cognitive theory consisting of four building blocks that already showed its potential to explain toxic behavior perpetration within the context of MOBAs (Xiao and Wong, 2013; Kordyaka et al., 2020).

First, the motivation toward toxic behavior (i.e., describing the maintenance of goal-directed behaviors) predicted the occurrence of toxic behavior perpetration (Kordyaka et al., 2020). We argue that due to the overlap in roles between perpetration and becoming a victim of toxicity, the motivation toward toxicity should be related to the likelihood of becoming a victim of toxic behavior. We justify this with the interdependence between being a perpetrator and becoming a victim of toxic behavior. Second, victimhood of toxicity already showed its potential to predict toxic behavior perpetration (Kordyaka et al., 2020). Complementary to this, we argue that past toxic behavior perpetration (i.e., the frequency with which a player exhibited toxic behavior in the past) support the occurrence of toxicity victimhood vice versa. For this, we refer to the cycle of violence hypothesis indicating that violent experiences in the past lead to involvement in comparable behaviors in the future (McCord, 1988). Third, self-efficacy (i.e., the self-evaluation of a player about capabilities to act in the ways necessary to reach specific goals) already showed its reducing influence on the occurrence of toxic behavior perpetration (Kordyaka et al., 2020). We want to extend this finding to the context of the experience of being a victim of toxicity. For this, we argue that players are aware of the detrimental influence of toxicity in relation to their performance. Accordingly, self-efficacy should have a negative influence on the occurrence of toxicity victimhood. Fourth, subjective norms (i.e., describing the belief that an important other will approve a particular behavior) showed insignificant results in previous research related to toxic behavior perpetration (Kordyaka et al., 2020). However, we still want to test its predictive potential for toxicity victimhood. For this, we argue that the perception of normative beliefs approving toxic behaviors of important others regarding toxic behavior increases the saliency of toxicity victimhood.

*Hypothesis SCT.1*: Motives toward toxic behavior perpetration have a positive influence on toxicity victimhood.

*Hypothesis SCT.2*: Toxic behavior perpetration has a positive influence on toxicity victimhood.

*Hypothesis SCT.3*: Self-efficacy toward toxic behavior has a negative influence on toxicity victimhood.

*Hypothesis SCT.4*: Subjective norms approving toxic behavior have a positive influence on toxicity victimhood.

#### 2.3.3. Theory of planned behavior

The theory of planned behavior is a widely applied cognitive psychological theory proposing that the execution of a specific behavior can be predicated by their intention to engage in that behavior (Ajzen, 1991, 2002). The theory already showed its potential to predict toxic behavior in MOBAs (Kordyaka et al., 2020). Furthermore, as antecedents of the behavioral intention the theory proposes three different antecedent variables which we seek to test in relation to toxicity victimhood.

First, we argue that attitude (i.e., the positive or negative evaluation) toward toxicity victimhood and a less severe evaluation of toxicity (a more positive attitude) increases the perception of being a victim of toxicity. Second, as the social cognitive theory, the theory of planned behavior proposes subjective norms (see Hypothesis SCT.4. for specific explanations) as a predictor variable. Third, regarding behavioral control (i.e., the perceived difficulty of performing a behavior), we argue that players who perceive toxic behavior as easier to control (i.e., have a higher efficacy of behavioral control) will show lower levels of toxic behavior because they are aware of the dysfunctional impact of toxicity on performance, which should lead to lower levels of toxicity victimhood as well. Based on this, we formulate the subsequent hypotheses related to the theory of planned behavior.

*Hypothesis TPB.1*: Attitude has a positive influence on toxicity victimhood.

*Hypothesis TPB.2*: Subjective norms have a positive influence on toxicity victimhood.

*Hypothesis TPB.2*: Behavioral control has a negative influence on toxicity victimhood.

## 3. Research methodology

### 3.1. Research design

**Figure 1:**
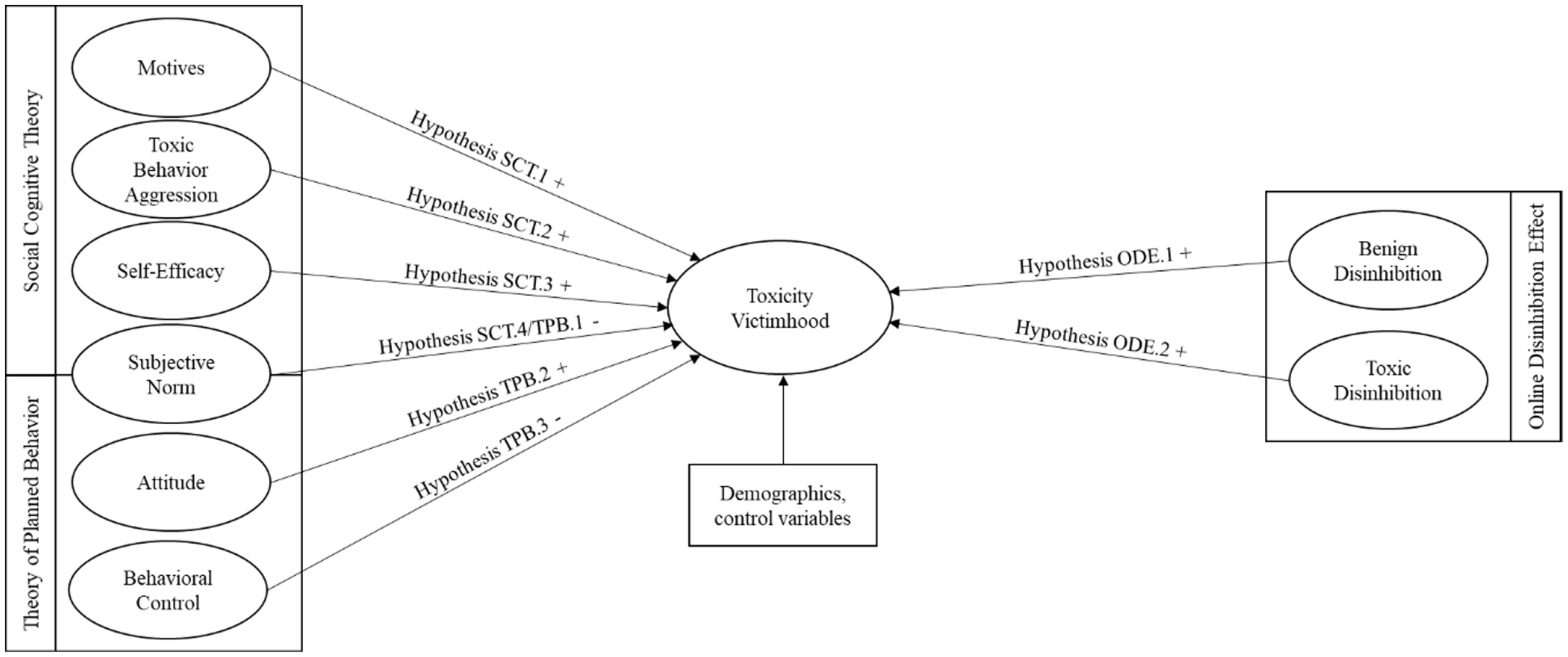
Research model.

For the purposes of our study, we used a cross-sectional survey approach and collected self-reported data from players using an online questionnaire. Methodologically, we analyzed the data with covariance-based statistics and structural equation modeling to explain toxicity victimhood, while controlling for demographic and control variables (see Figure 1). To derive our quantitative results, we used the software packages SPSS 28 and AMOS 28.

### 3.2. Data collection and participants

Initially, survey responses consisted of 320 participants using the crowdsourcing marketplace Mechanical Turk (MTurk). Each participant received $1.89 for participating in our study. First, we excluded four participants who reported inconclusive demographic answers (such as playing 0 h a week) or had missing values. Second, to ensure that the participants followed the requirement of playing either League of Legends or Defense of the Ancients 2, we asked them to specify their three most favorite in-game characters in an open text field. After inspecting the answers, we excluded three more participants. Accordingly, the final sample consisted of 313 participants.

Most participants were male (209 male, 104 female) and had an average age of 29 years (*M* = 29.18, SD = 6.91). Most participants were Americans (157) and stated that they had finished their bachelor’s degree (80%). Additionally, most participants reported that they used a personal computer as their primary game playing device (58%), been playing Defense of the Ancients2 or League of Legends for a little more than 6 years (*M* = 6.33, SD = 5.67) around 8 h a week (*M* = 8.71, SD = 8.99). In addition, 171 participants specified that they predominantly play Defense of the Ancients 2, while 142 participants predominantly played League of Legends. Summarizing, the demographic characteristics of our sample seemed to be representative in relation to the ordinary players in previous research (Kordyaka et al., 2020).

### 3.3. Operationalization of variables

To measure the constructs of our study, we used validated scales and items from previous research adjusted to the context of our study as necessary. Most scales used a seven-point Likert scale ranging from 1 (“strongly disagree”) to 7 (“strongly agree”). All items used in our study are included in the appendix (see Appendix Table 1). Additionally, we collected demographic variables (e.g., age, sex, education, and country) and control variables (e.g., hours of play, experience of play, platform, and game^1^) to further prevent unwanted confounding influences.

## 4. Results

### 4.1. Validation of the measurement instrument

To derive validity indicators of our measurement models for all three theoretical approaches, we assessed convergent and discriminant validity. For convergent validity, we used the composite reliability (CR) and the average variance extracted (AVE) (Gefen et al., 2000). To test discriminant validity, we used the Fornell-Larcker criterion, which postulates that a measurement model is supported when the square root of the AVE of each construct is greater than the correlations between each construct and the other constructs and checked for factor loadings and cross-loadings (Fornell and Larcker, 1981; Table 1).

**Table 1:** Descriptive statistics and construct correlations.

			CR	Mean	SD	1	2	3	4	5
ODE	1	Benign disinhibition	0.84	5.12	1.04	0.72				
	2	Toxic disinhibition	0.89	4.06	1.68	0.31^***^	0.82			
	3	TB victimhood	0.93	5.06	1.22	0.44^***^	0.39^***^	0.77		
SCT	1	Motives	0.80	4.84	1.30	0.75				
	2	TB perpetration	0.93	4.92	1.23	0.44^***^	0.86			
	3	Self-efficacy	0.87	5.42	1.01	0.31^***^	−0.04	0.73		
	4	Subjective norms	0.76	5.27	1.30	0.09	−0.08	0.52^***^	0.78	
	5	TB victimhood	0.86	5.06	1.22	0.38^***^	0.33^***^	0.35^***^	0.20^***^	0.77
TPB	1	Attitude	0.96	3.80	2.04	0.90				
	2	Subjective norms	0.82	5.27	1.31	−0.10	0.83			
	3	Behavioral control	0.86	5.18	1.50	−0.19^**^	0.34^***^	0.87		
	4	TB victimhood	0.81	5.06	1.22	0.28^***^	0.20^***^	0.28^***^	0.80	

(a) CR: Composite reliability; (b) Diagonal elements are the square root of the shared variance between the constructs and their measures; (c) Off-diagonal elements are correlations between constructs; ***p < 0.001, **p < 0.01.

#### 4.1.1. Online disinhibition effect

Following the previously described procedure, we carried out a principal component analysis using varimax rotation. Additionally, we specified the extraction of three factors and inserted seven items of benign disinhibition, four items of toxic disinhibition, and five items of toxicity victimhood. After inspecting the initial results, we excluded two of the benign disinhibition items (i.e., BD_5 “I have an image of the other players in my head when I read their messages” and BD_6 “I feel like a different person online”) and one of the toxicity victimhood items (TBV_1 “…intentionally interrupt me while I am writing”) because the item showed low and/or unclear loading patterns. After the item exclusion, all composite reliabilities exceeded 0.7 (≥ 0.84), the AVE of each construct was greater than 0.5 (≥0.51), and all items loaded on the intended factors ( |≥0.66| ). Accordingly, convergent validity was satisfied. Additionally, the square root of the AVE of each construct (≥0.72) was greater than the correlations between each construct and the other constructs (≤0.44), and no meaningful cross-loadings were found satisfying the conditions for discriminant validity.

#### 4.1.2. Social cognitive theory

To test the measurement model of the social cognitive theory, we used a principal component analysis using varimax rotation specifying the extraction of five factors and inserted three motive items, five toxic behavior perpetration items, six items of self-efficacy, three items of subjective norms, and four items of toxicity victimhood (based on the previous finding). After inspecting the initial results, we excluded one of the subjective norms items (i.e., SN_2 “I think players who matter to me would appreciate it if I assisted a toxic player”), because the item showed unclear loading patterns. After the item exclusion, all composite reliabilities exceeded 0.7 (≥0.76), the AVE of each construct was greater than 0.5 (≥0.54), and all items loaded on the intended factors ( |≥0.65| ). Accordingly, convergent validity seemed satisfied. Furthermore, the square root of the AVE of each construct (≥0.73) was greater than the correlations between each construct and the other constructs (≤0.52), and no meaningful cross-loadings were found satisfying the conditions for discriminant validity.

#### 4.1.3. Theory of planned behavior

Using the previously described procedure, we specified the extraction of four factors and inserted six items of attitude, two items of subjective norm (based on the previous finding), four items of behavioral control, and four items of toxicity victimhood. After inspecting the initial results, we excluded the two of the behavioral control items (i.e., BC_2 “It is very difficult” and BC_4 “I am very likely to fail”), because both items showed unclear loading patterns. After the exclusion of both items, all composite reliabilities exceeded 0.7 (≥0.81), the AVE of each construct was greater than 0.5 (≥0.63), and all items loaded on the intended factors ( |≥0.75| ). In addition, the square root of the AVE of each construct (≥0.80) was greater than the correlations between each construct and the other constructs (≤0.34), and no meaningful cross-loadings were found satisfying the conditions for discriminant validity.

### 4.2. Theory tests

#### 4.2.1. Online disinhibition effect

In case of the online disinhibition effect, we specified benign and toxic disinhibition and the demographic (age, sex, education, and country) and control variables (hours of play, experience of play, platform, and game) as independent variables to explain the dependent variable toxicity victimhood. The regression equation showed a significant result (*F* (10,302) = 12.17, *p* < 0.001) and explained 26% of the variance of toxicity victimhood. Furthermore, benign disinhibition (*β* = 0.37, *p* < 0.001) and toxic disinhibition (*β* = 0.29, *p* < 0.001) showed significant influences opposed to all demographic and control variables (all others *p* ≥ 0.06). Accordingly, we interpret both results as empirical support for both hypotheses related to the online disinhibition effect (hypothesis ODE.1 “Benign disinhibition has a positive influence on toxicity victimhood” and hypothesis ODE.2: “Toxic disinhibition has a positive influence on toxicity victimhood”), while controlling for potential confounds of demographic and control variables.

#### 4.2.2. Social cognitive theory

With regard to the social cognitive theory, we used toxic behavior perpetration, self-efficacy, motives, subjective norm, and the demographic (age, sex, education, and country) and control variables (hours of play, experience of play, platform, and game) as independent variables to explain the dependent variable toxicity victimhood. The regression equation showed a significant result (*F* (12,300) = 10.24, *p* < 0.001) and explained 26% of the variance of toxicity victimhood. Additionally, toxic behavior perpetration (*β* = 0.30, *p* < 0.001), self-efficacy (*β* = 0.27, *p* < 0.001), motives (*β* = 0.19, *p* < 0.01), and the control variable experience of play (*β* = −0.11, *p* < 0.05) showed significant influences opposed to subjective norms (*β* = 0.08, *p* = 0.20) and all other variables (*p* ≥ 0.20). Accordingly, we understand the results as empirical support for two of the four hypotheses related to the social cognitive theory (hypothesis SCT.1: Motives toward toxicity have a positive influence on toxicity victimhood, hypothesis SCT.2: Toxic behavior perpetration has a positive influence on toxicity victimhood), while controlling for potential confounds of demographic and control variables. Contrary to our hypothesized relationships in hypothesis SCT.3 self-efficacy had a positive influence of toxicity victimhood and subjective norms did not reach the necessary significancy threshold.

#### 4.2.3. Theory of planned behavior

In case of the theory of planned behavior, we inserted attitude, subjective norm, behavioral control, and the demographic (age, sex, education, and country) and control variables (hours of play, experience of play, platform, and game) to explain the dependent variable toxicity victimhood. The regression equation showed a significant result (*F* (11,301) = 5.68, *p* < 0.001) and explained 14% of the variance of toxicity victimhood. Moreover, attitude (*β* = 0.24, *p* < 0.001), subjective norm (*β* = 0.18, *p* < 0.01), and behavioral control (*β* = 0.17, *p* < 0.001) showed significant influences (all others *p* ≥ 0.12). Based on this, we found empirical support for all three hypotheses related to the theory of planned behavior (hypothesis TPB.1: attitude has a positive influence on toxicity victimhood, hypothesis TPB.2: subjective norms have a positive influence on toxicity victimhood, hypothesis TPB.3: behavioral control has a positive influence on toxicity victimhood), while controlling for potential confounds of demographic and control variables.

#### 4.2.4. Comparison of theories

To compare the explanatory potential of all three theories (i.e., online disinhibition effect, social cognitive theory, theory of planned behavior) in relation to toxicity victimhood, Table 2 illustrates the results:

**Table 2:** Comparison of theories explaining toxicity victimhood.

Variable	Model 1 (ODE)	Model 2 (SCT)	Model 3 (TPB)
Age	0.03	0.01	−0.02
Sex	0.05	0.07	0.08
Education	0.02	−0.03	0.02
Country	−0.06	−0.05	−0.03
Hours a week	−0.04	−0.04	0.01
Experience of play	−0.10	−0.11^*^	−0.07
Platform	−0.04	−0.06	0.01
Game	−0.02	0.01	0.05
Benign disinhibition	0.37^***^		
Toxic disinhibition	0.29^***^		
Motives		0.19^**^	
TB perpetration		0.30^***^	
Self-efficacy		0.27^***^	
Subjective norm		0.08	0.18^**^
Attitude			0.24^***^
Behavioral control			0.17^**^
*R* ^2^	0.29	0.29	0.17
*R*^2^ adjusted	0.26	0.26	0.14

***p < 0.001; **p < 0.01; *p < 0.05.

Based on our results, we see that constructs related to the online disinhibition effect (benign and toxic disinhibition) and the social cognitive theory (motives, toxic behavior perpetration, self-efficacy, and subjective norm) have the same explanatory potential (26%). However, based on the demand for parsimony, we argue that the online disinhibition effect is the most appropriate approach to better understand toxicity victimhood.

### 4.3. Unifying the theories

To derive a unified model accounting for variables of all three theories, we proceeded in five subsequent steps. First, we carried out three multiple regression analyses inserting demographic (age, sex, education, country) as well as control variables (i.e., hours played, experience of play, platform, and game) as independent variables explaining the dependent variable toxicity victimhood to control for potential confounding influences. The regression equation did not indicate a relevant influence (*F* (8,304) = 1.56, *p* = 0.14) and none of the predictor weights showed a significant influence (*p* ≥ 0.07). Consequently, we noted that we did not have to consider any of the demographic or control variables within our subsequent analytical steps.

Second, we wanted to understand direct influences of the variables of the three theories in relation to toxicity victimhood, while examining all potential antecedents simultaneously. Accordingly, we used a multiple regression analysis using benign and toxic disinhibition, toxic behavior perpetration, self-efficacy, motives, attitude, subjective norms, and behavioral control as predictors of toxicity victimhood. The regression equation showed a significant result (*F* (8,304) = 16.67, *p* < 0.001) and explained 29% of the variance and benign (*β* = 0.18, *p* < 0.05) and toxic disinhibition (*β* = 0.18, *p* < 0.05), as well as self-efficacy (*β* = 0.15, *p* < 0.05) had significant influences (all others *p* ≥ 0.12).

Third, we searched for predictors of benign disinhibition. For this, we used a multiple regression analysis including toxic disinhibition, toxic behavior perpetration, self-efficacy, motives, attitude, subjective norms, and behavioral control as predictors of benign disinhibition. The regression equation showed a significant result (*F* (7,305) = 56.13, *p* < 0.001) and explained 55% of the variance of benign disinhibition. Additionally, toxic disinhibition (*β* = 0.16, *p* < 0.05), self-efficacy (*β* = 0.47, *p* < 0.001), motives (*β* = 0.36, *p* < 0.001), and attitude (*β* = −0.18, *p* < 0.01) had significant influences explaining benign disinhibition (all others *p* ≥ 0.13).

Fourth, we wanted to identify predictors of toxic disinhibition. For this, we used a multiple regression analysis including the variables benign disinhibition, toxic behavior perpetration, self-efficacy, motives, attitude, subjective norms, and behavioral control as predictors of toxic disinhibition. The regression equation showed a significant result (*F* (7,305) = 92.08, *p* < 0.001) and explained 67% of the variance of toxic disinhibition. Furthermore, benign disinhibition (*β* = 0.12, *p* < 0.05), toxic behavior perpetration (*β* = 0.45, *p* < 0.001), and attitude (*β* = 0.37, *p* < 0.001) had significant influences explaining toxic disinhibition (all others *p* ≥ 0.12).

**Figure 2:**
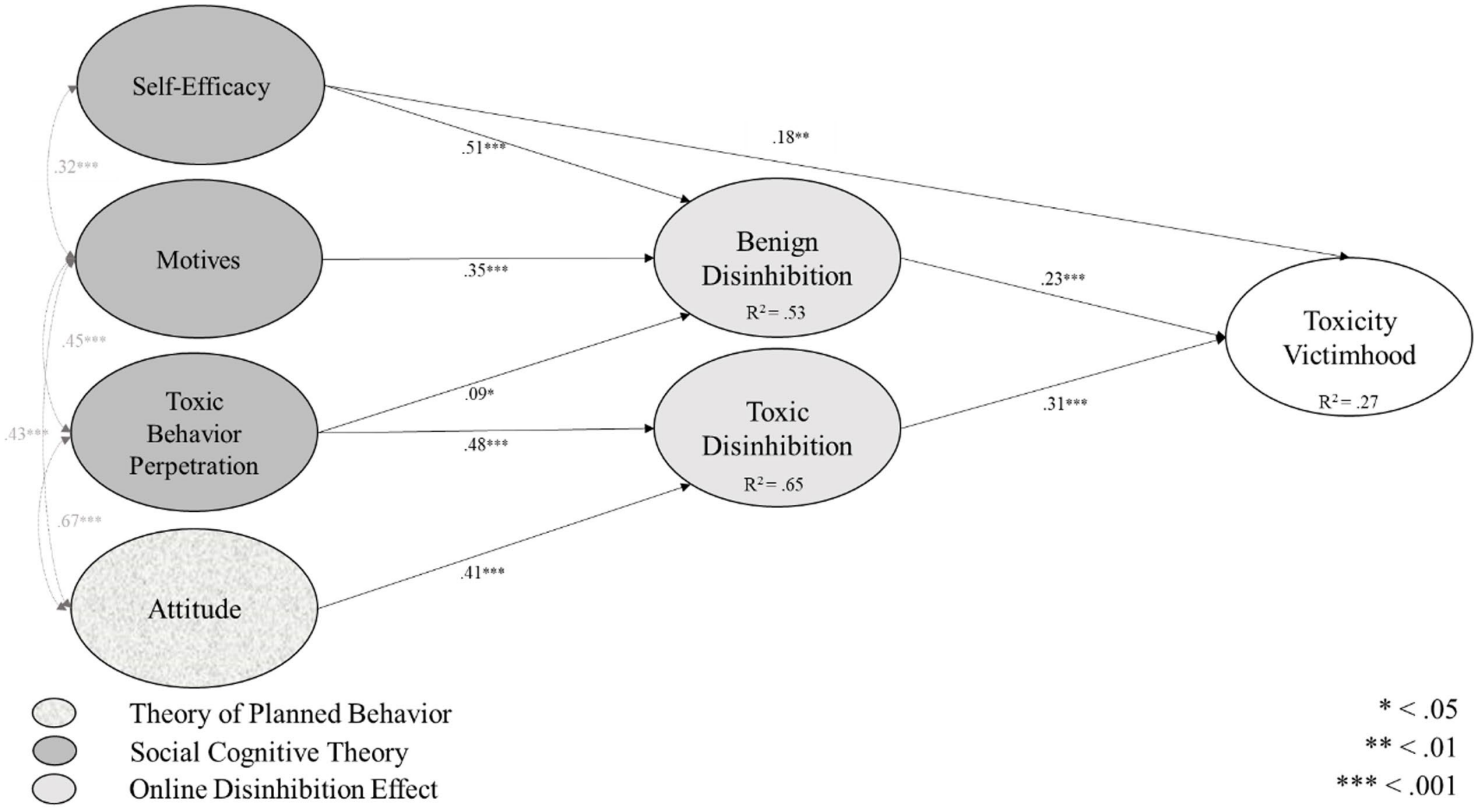
Unified theory of toxic behavior victimization.

Fifth, we used the derived information and inserted the identified relationships into a structural equation (path) model (Kline, 2015). The results of the path model only showed little room for improvement (*χ*^2^ (2,320) = 23.66, *p* = 0.1). However, the significance test of the model is no longer relied upon as a basis for acceptance or rejection of a model (Schermelleh-Engel et al., 2003; Vandenberg, 2006). Therefore, we assessed additional fit values, which consistently indicated an excellent fit between the theoretical model and the empirical data (CFI = 0.98, RMSEA = 0.07, SRMR = 0.03). Additionally, all predictors accounted for 27% of the variance of toxicity victimhood, 53% of benign and 65% of toxic disinhibition. On a level of content, self-efficacy (*β* = 0.18, *p* < 0.01), benign (*β* = 0.23, *p* < 0.001), and toxic disinhibition (*β* = 0.31, *p* < 0.001) significantly influenced toxicity victimhood. Additionally, self-efficacy (*β* = 0.51, *p* < 0.001), motives (*β* = 0.35, *p* < 0.001), and toxic behavior perpetration (*β* = 0.09, *p* < 0.05) predicted benign disinhibition, while toxic behavior perpetration (*β* = 0.48, *p* < 0.001) and attitude (*β* = 0.41, *p* < 0.001) showed significant influences on toxic disinhibition. Additionally, we allowed for correlations across independent variables (see Figure 2).

## 5. Discussion

### 5.1. Main findings

Based on the findings of our study, we can now provide an empirically based answer to our research question—What variables informed by theory explain the experience of becoming a victim of toxicity in multiplayer online battle arena games? Consequently, we summarize our key findings with the following three points:

First, we explored the explanatory potential of three well-established theoretical approaches (online disinhibition effect, social cognitive theory, and theory of planned behavior) in relation to the experience of being a victim of toxicity. Specifically, we showed that the variables benign disinhibition, toxic disinhibition, and self-efficacy together shape toxicity victimhood. Having a substantiated type of generalized knowledge about the emergence of toxicity victimhood adds value to existing research related to toxic behavior in general.

Second, we showed that the most relevant predictors of the experience of being a victim of toxicity were the two online disinhibition variables benign and toxic disinhibition. This finding adds to existing research that already explained toxic behavior perpetration (Kordyaka et al., 2020) adding relationships that are specific to the role of becoming a victim of toxicity. Opposed to the case of toxic behavior perpetration, benign disinhibition was a relevant antecedent of the experience of being a victim of toxicity indicating a more complex emergence. Third, based on our results, self-efficacy was the most relevant antecedent variable of benign disinhibition. This finding is an indicator that the self-evaluation has the capability to adequately deal with toxicity and enables the perception of higher levels of benign disinhibition. We interpret this finding in a way that players with higher levels in self-efficacy have less worries about sharing personal information, which should lead to higher levels of benign disinhibition. Additionally, toxic behavior perpetration was the most relevant antecedent for toxic disinhibition suggesting that the own execution of toxic behavior in the past lead to higher levels of toxic disinhibition based on the more ordinary perception of corresponding behaviors (Fox and Tang, 2014).

### 5.2. Implications for theory

By closing the research gap of a missing theoretical explanation of the experience of being a victim of toxicity using three well-established theoretical frameworks (i.e., online disinhibition effect, social cognitive theory, and theory of planned behavior), the results of our study allow for several implications in relation to existing theoretical work dealing with toxic behavior. Subsequently, we will elaborate on them in relation to the three theoretical approaches of our study (i.e., online disinhibition effect, social cognitive theory, and theory of planned behavior).

#### 5.2.1. Related to the online disinhibition effect

First, our results underlined the prominent relevance of the online disinhibition effect in the context of toxic behavior in an unambiguous manner adding complementary insights regarding the experience of being a victim of toxicity. On the one hand, our insights increased the external validity (Kordyaka and Kruse, 2021) of the influence of toxic disinhibition by showing a positive relationship to toxicity victimhood empirically supporting our hypothesis ODE.2 (“toxic disinhibition has a positive influence on toxicity victimhood”). Accordingly, and in line with previous research, toxic disinhibition reduces personal responsibility, increase anonymity, and facilitates social comparison, all of which can contribute to the occurrence of toxic behavior. We understand this finding as an indicator of a substantial overlap in the roles of perpetrators and victims in the MOBA context. On the other hand, opposed to existing work regarding toxic behavior perpetration (Sengün et al., 2019; Kordyaka et al., 2020), benign disinhibition was a particularly relevant and positive predictor of toxicity victimhood empirically supporting our hypothesisODE.1 (“benign disinhibition has a positive influence on toxicity victimhood”). We explain this finding because benign disinhibition relates to the absence of restraint while playing, which might increase the sharing of personal information that provide perpetrators of toxicity with potential points of references for their toxic perpetration. Nonetheless, we encourage future research to explore the relationship between toxic and benign disinhibition as antecedents of the emergence of toxicity in more detail.

#### 5.2.2. Related to social cognitive theory

Second, the results of our study in relation to the social cognitive theory add value to existing theoretical knowledge providing another elementary building block to better comprehend the emergence of toxicity victimhood. On the one hand, as postulated, motives (hypothesis SCT.1 “motives toward toxic behavior perpetration have a positive influence on toxicity victimhood”) and toxic behavior perpetration (hypothesis SCT.2 “toxic behavior perpetration has a positive influence on toxicity victimhood.”)explained the experience of being a victim of toxicity in a positive manner. We argue that both findings support the assumption of a substantial overlap in roles of becoming a perpetrator and a victim of toxicity that has already been indicated in previous research (Kordyaka et al., 2020) as well as in the original theoretical context of cyberbullying research (Baldry et al., 2017; Estévez et al., 2020). On the other hand, subjective norms (hypothesis SCT.4) did not show a relevant influence on toxicity victimhood, which is in line with previous work related to toxicity perpetration (Kordyaka et al., 2020). Furthermore, opposed to our hypotheses, self-efficacy (hypothesis SCT.4) showed a positive influence on toxicity victimhood, which was a surprising result. A potential explanation for this could be that self-efficacy toward toxicity may increase victimhood if it leads to maladaptive coping strategies. For example, if a player believes they have the skills to handle toxic behavior, but instead engages in constant confrontations with other players, it can perpetuate a cycle of victimhood by repeatedly engaging in toxic interactions without seeking healthier solutions. In such cases, self-efficacy may lead to an overreliance on confrontational or avoidant behaviors, rather than fostering assertive communication, boundary-setting, and problem-solving skills. However, this is just one potential explanation and future research is clearly needed.

#### 5.2.3. Related to theory of planned behavior

Finally, regarding the theory of planned behavior, attitude (hypothesis TPB.1), subjective norm (hypothesis TPB.2), and behavioral control (hypothesis TPB.3), all showed significant influences on the experience of being a victim of toxicity. This is partly in line with previous work regarding toxic perpetration (Kordyaka et al., 2020). On the one hand, the positive influence of attitude on being a victim of toxicity increases the number of indicators regarding the overlap between toxicity perpetration and victimhood. On the other hand, and opposed to toxicity perpetration, subjective norms and behavioral control had significant and positive influences on the occurrence of toxicity victimhood. We understand the positive influence of subjective norm on toxicity victimhood as a reference to the saliency and marginalization of toxicity that can be partly attributed to the circumstance that toxicity is an oftentimes accepted part of the game-related culture (Sengün et al., 2019; Beres et al., 2021). Additionally, and opposed to our hypotheses, behavioral control had a positive influence on toxicity victimhood. One potential explanation for this could be that players who exercise behavioral control refraining from responding to toxic behavior may perceive themselves victimized or disadvantaged, as they may perceive themselves as not using the same strategies as toxicity perpetrators, and this could lead to an increased perception of toxicity victimhood. However, this is only one potential explanation and we understand this result as an indication of the complexity of the immanent interactions between the roles related to toxicity.

### 5.3. Implications for practice

Toxic behavior is one of the biggest challenges for the present industry of MOBAs because players experiencing toxicity may choose to leave the game or initiate more toxicity in return. Consequently, toxicity leads to substantial loss of revenue. Thus, an adequate handling of toxic behavior is critical for the future success of MOBAs. Our findings provide points of reference to better deal with the emergence of toxicity providing points of reference to better understand the experience of being a victim of toxicity. In the following, we discuss them in relation to our three theoretical approaches (i.e., online disinhibition effect, social cognitive theory, and theory of planned behavior).

#### 5.3.1. Related to the online disinhibition effect

First, based on our findings related to the online disinhibition effect, both (benign and toxic) dimensions of online disinhibition were meaningful (positive) predictors of the experience of being a victim of toxicity. We interpret both findings as a call to undertake actions for game developers and publishers to try to reduce the perceptions of disinhibition to decrease the likelihood of their player bases experiencing toxic behavior. Potentially fruitful avenues could be to use techniques of real-world identity proofing requiring players to provide basic identifying information such as legal name, date of birth, or place of residency when downloading the relevant MOBA or the fostering of positive behavioral incentives to decrease the likelihood of toxicity to emerge. Additionally, design interventions could comprise real-time feedback to reduce disinhibition during gameplay and break the cycle of toxicity during games, which is an intervention Kordyaka and Kruse (2021) already proposed to tackle the emergence of toxic behavior perpetration.

#### 5.3.2. Related to social cognitive theory

Second, the explored information regarding the social cognitive theory includes points of reference to better handle toxic behavior in practice. Specifically, motives, toxic behavior perpetration, and self-efficacy showed relevant influences. As a response to this, based on the indicated overlap of roles of toxicity perpetration and victimhood, educational programs could be used underlining the detrimental consequences of toxicity negatively affecting performance and the well-being of players (Kowert, 2020; Monge and O’Brien, 2022) that should reduce online disinhibition and the likelihood of the emergence of toxicity. To ensure an adequate learning success, negative (e.g., loss of points, restrictions) as well as positive design features (e.g., social rewards, and specific skins) within the game could be used. Furthermore, the identified increasing influence of self-efficacy on the experience of being a victim of toxicity could be addressed by providing players with accurate feedback encouraging self-reflection communication strategies comprising information to educate players about the detrimental value of toxicity preventing a marginalization of cascading influences of toxicity.

#### 5.3.3. Related to the theory of planned behavior

Third, findings related to the theory of planned behavior include added-value for practice as well. Specifically, the positive influences of attitude and subjective norms on the experience of being a victim of toxicity indicate potential for the industry of MOBAs. In relation to both findings, we recommend to get measures underway that reduce the (rather) accepted player evaluation of toxicity in MOBAs underlining the detrimental consequences of toxicity (Fox and Tang, 2014; Beres et al., 2021). One potential point of reference could be to approach the underlying subjective norms with the aid of social learning (Bandura, 1986). Specifically, the industry could work together with well-known personalities from the MOBA game genre (such as professional players, coaches, and casters) promoting positive role modeling to exemplify desired behaviors and call attention to the detrimental influences of toxic behavior. Another avenue would be to derive assistant systems that help players to better understand the negative consequences of toxic behavior and deteriorate their opinion about toxicity.

### 5.4. Limitations and outlook

As with every empirical study, this study is not without some limitations that we had to accept on the grounds of research economy. First, our sample consisted only of English-speaking participants. Thus, future research is needed to determine whether our results can be generalized to other regions and cultures and cross-cultural research is needed that has been noted in previous work related to MOBAs already (Kordyaka et al., 2022). Additionally, we used M-Turk as a source of data, which might have confounded our results. To have the chance to control for such influences we encourage future research to collect field data using their internal and external networks and compare their results to the results of our study. Second, despite the fact we carried out an additional analysis to derive indicators of the validity of our measurement instrument of toxicity victimhood comparing self-reported and behavioral data, we suggest that it would add value if studies would investigate this aspect in more detail. One fruitful avenue could be to substantially increase the sample size, which could even be achieved by implementing tools of artificial intelligence and machine learning to code large quantities of behavioral data detecting behavioral patterns of players (Idalski Carcone et al., 2019). Third, with our methodological approach, we were only able to identify correlational relationships between the variables of our study. This was intended and we just wanted to provide initial indicators regarding an explanation of the experience of being a victim of toxicity. Taking our approach one step further, future research could build upon our findings to test the relationships of the emergence of toxicity contrasting different roles toward causality using field experiments (Harrison and List, 2004). Fourth, we only looked at a specific game genre not including a wide variety of video games, which was intended to avoid potential confounds. However, we strongly recommend to test the insights of our study in neighboring (multiplayer) game genres such as location-based games that provide a rich and connectable portfolio of current research work (Laato et al., 2021, 2022). Fifth, as part of our study, we only included the biological sex of participants to control for potential confounds (Kordyaka and Brunnhofer, 2021). To obtain a richer picture at this point, we suggest to include aspects related to gender in future research to explore the representation and portrayal of male and female players in the game, as well as the potential impact of gender norms and stereotypes on player behavior and community dynamic.

## 6. Conclusion

In this research, we have pursued the goal to find an explanation for the emergence of the experience of being a victim of toxicity in MOBA games. Drawing from the theoretical frames of the online disinhibition effect, social cognitive theory, and theory of planned behavior, we carried out a quantitative survey approach. Our findings revealed that benign and toxic disinhibition were the most relevant antecedent variables of toxicity victimhood. Additionally, opposed to our hypotheses, higher levels of self-efficacy predicted becoming a victim of toxicity more frequently indicating complex underlying mechanisms. Through these insights, we add to the knowledge related to the emergence of toxic behavior by providing variables that can be considered in an evidence-based manner when looking into strategies to mitigate toxicity.

## Data availability statement

The original contributions presented in the study are included in the article/Supplementary material, further inquiries can be directed to the corresponding author.

## Ethics statement

Ethical review and approval was not required for the study on human participants in accordance with the local legislation and institutional requirements. The patients/participants provided their written informed consent to participate in this study. All data collected was kept confidential and only used for the purpose of this study. No personally identifiable information was collected.

## Author contributions

BK writing original draft and formal analysis. SL writing original draft. SW review and editing. BN review, editing, and supervision. All authors contributed to the article and approved the submitted version.

## Conflict of interest

The authors declare that the research was conducted in the absence of any commercial or financial relationships that could be construed as a potential conflict of interest.

## Publisher’s note

All claims expressed in this article are solely those of the authors and do not necessarily represent those of their affiliated organizations, or those of the publisher, the editors and the reviewers. Any product that may be evaluated in this article, or claim that may be made by its manufacturer, is not guaranteed or endorsed by the publisher.

## Footnotes

^1^A nominal factor comprising the two MOBAs League of Legends and Defense of the Ancients 2.
