# Supplementary material for: What constitutes victims of toxicity - identifying drivers of toxic victimhood in multiplayer online battle arena games
Source: Front Psychol. 2023 Jun 16;14:1193172. doi: 10.3389/fpsyg.2023.1193172 (PMC10313333; doi:10.3389/fpsyg.2023.1193172)
Supplement: Supplementary file 1 [file Table_1.docx]

| **Table Appendix 1.** Questionnaire items | | | | |
| --- | --- | --- | --- | --- |
| Construct |  | Wording | Reference | |
| **TB** |  | “If others get mad at me during a game, they…” | |  |
| Experience of being a victim | TV_1 | “...intentionally interrupt me while I am writing.” | | (Kordyaka, Jahn, und Niehaves 2020) |
|  | TV _2 | ”...hold me responsible making own mistakes.” | |  |
|  | TV _3 | “...take away resources belonging to me.” | |  |
|  | TV _4 | “...insult me.” | |  |
|  | TV _5 | “...criticize me.” | |  |
|  |  |  |  | |
| **ODE** |  | “Please indicate how much you agree with the statements regarding the play of League of Legends/Defense of the Ancients 2” |  | |
| Benign  disinhibition | BD_1 | “It is easier to connect with others through the game than talking in person.” | (Udris 2014) | |
|  | BD_2 | “The game is anonymous, so it is easier for me to express my true feelings or thoughts.” |  |  |
|  | BD_3 | “It is easier to write things during a game that would be hard to say in real life because you don’t see the other’s face.” |  |  |
|  | BD_4 | “It is easier to communicate during a game because you can reply anytime you like.” |  |  |
|  | BD_5 | “I have an image of the other players in my head when I read their messages.” |  |  |
|  | BD_6 | “I feel like a different person during a game.” |  |  |
|  | BD_7 | “I feel that during games I can communicate on the same level with others who are older or have higher status.” |  |  |
|  |  |  |  | |
| Toxic disinhibition | TD_1 | “I don’t mind writing insulting things about others online because it’s anonymous” | (Udris 2014) | |
|  | TD_2 | “It is easy to write insulting things online because there are no repercussions.” |  |  |
|  | TD_3 | “There are no rules online therefore you can do whatever you want.” |  |  |
|  | TD_4 | “Writing insulting things online is not TB [R].” |  |  |
|  |  |  |  | |
| **SCT** |  | “Please indicate how much the subsequent motives apply to you as a person while playing the specified game” |  | |
| Motives for action | M_1 | “Power” | (Reiss 2004) | |
|  | M_2 | “Independence” |  | |
|  | M_3 | “Status” |  | |
|  |  |  |  | |
| Tendency for toxicity |  | “If I get mad during a game, I…” | (Kordyaka, Klesel, und Jahn 2019b) | |
|  | TP_1 | “...intentionally interrupt others while they are writing.” |  |  |
|  | TP _2 | ”...hold others responsible making own mistakes.” |  |  |
|  | TP _3 | “...take away resources belonging to others.” |  |  |
|  | TP _4 | “...insult others.” |  | |
|  | TP _5 | “...criticize others.” |  | |
|  |  |  |  | |
| Self-efficacy |  | “I feel confident…” | (Hsu and Chiu, 2004) | |
|  | SE_1 | “...completing my tasks while playing a game.” |  |  |
|  | SE_2 | “...visiting the store for buying items.” |  |  |
|  | SE_3 | “...navigating over the map using pings during a game.” |  |  |
|  | SE_4 | “...knowing information about the most recent changes/patches in the game.” |  |  |
|  | SE_5 | “...knowing information about the most recent patches and software changes.” |  |  |
|  | SE_6 | “…understanding the calls to action of other players during a game.” |  |  |
|  |  |  |  | |
| Subjective norms | SN_1 | “Most players who are important to me do not perpetrate TB.” | (Venkatesh u. a. 2003) | |
|  | SN_2 | “I think players who matter to me would appreciate it if I assist a toxic player [R].” |  | |
|  | SN_3 | “Players who influence my behavior think I should not exhibit TB.” |  | |
|  |  |  |  | |
| **TPB** |  |  |  | |
| Subjective norms |  | See SCT |  | |
|  |  |  |  | |
| Positive attitude towards toxicity |  | ” I perceive Toxic Behavior as:” | (Heirman und Walrave 2012) | |
|  | A_1 | “bad – good” |  |  |
|  | A_2 | “foolish – wise” |  |  |
|  | A_3 | “dislike – like” |  |  |
|  | A_4 | “unpleasant – pleasant” |  |  |
|  | A_5 | “harmful – not harmful” |  |  |
|  | A_6 | “disadvantageous – advantageous” |  |  |
|  |  |  |  | |
| Behavioral control |  | “When I want to prevent myself from exhibiting TB…” | (de Bruijn et al., 2009) | |
|  | BC_1 | “...it is very easy.” |  |  |
|  | BC_2 | “...it is very difficult [R].” |  |  |
|  | BC_3 | “...I am very likely to succeed.” |  |  |
|  | BC_4 | “...I am very likely to fail [R].” |  |  |

*[R] describe reverse coded items.*
